# A simplified clinical frailty scale predicts mortality in emergency department patients with acute dyspnea

**DOI:** 10.1007/s11357-025-01864-7

**Published:** 2025-09-11

**Authors:** Ahmad Zwawi, Torgny Wessman, Per Wändell, Olle Melander, Axel C. Carlsson, Toralph Ruge

**Affiliations:** 1 Department of Emergency and Internal Medicine, Skåne University Hospital, Malmö, Sweden; 2 Department of Clinical Sciences, Malmö, Lund University & Department of Internal Medicine, Skåne University Hospital, Malmö, Sweden; 3 Department of Neurobiology, Care Sciences and Society, Karolinska Institutet, Huddinge, Sweden; 4 Center for Primary Health Care Research, Lund University, Malmö, Sweden; 5 Academic Primary Care Center, Region Stockholm, Sweden

**Keywords:** Dyspnea, Frailty, Aging, Mortality, ED

## Abstract

To evaluate a simplified version of the Clinical Frailty Scale (SCFS) among older adults presenting to the emergency department (ED) with acute dyspnea. In this retrospective single-center cohort study, we included patients from the Acute Dyspnea Study (ADYS) cohort. Severity of illness was assessed using the Medical Emergency Triage and Treatment System (METTS). SCFS was operationalized using existing data on municipal care services from the ADYS database and divided into three levels. SCFS 1: Not frail patients with no need for municipal care services, SCFS 2: Patients with municipal care services, including home care, and SCFS 3: Patients with residence in a short-term care facility or nursing home. The primary outcome was 90-day mortality and hospitalization. Multivariable Cox and logistic regression analyses were used to assess associations between SCFS and outcome variables. SCFS criteria were met in 35.2% of patients (668 patients, SCFS group 2 and 3). These individuals had a higher comorbidity burden and increased 90-day mortality (20.9%, p < 0.001). SCFS group 3 was independently associated with a higher risk of 90-day mortality (HR = 2.60, 95% CI: 1.27–5.29, p = 0.009), compared to group 1. ROC curve analysis showed that combining SCFS with METTS significantly improved predictive performance (DeLong’s test: p = 0.015 and p = 0.0322 in respective models). For hospitalization, SCFS group 3 was associated with hospitalization independent of age, sex, BMI, comorbidities, and readmission, when compared to SCFS group 1 (OR = 2.57, CI:1.11–6.71, p = 0.037). This association was attenuated and nonsignificant after further adjustment for METTS. SCFS is an independent predictor of 90-day mortality in older ED patients with acute dyspnea. When combined with triage scores like METTS, its predictive value improves. These findings support the potential clinical utility of incorporating frailty assessment into ED triage to aid risk stratification and guide care decisions.

**Graphical Abstract:**

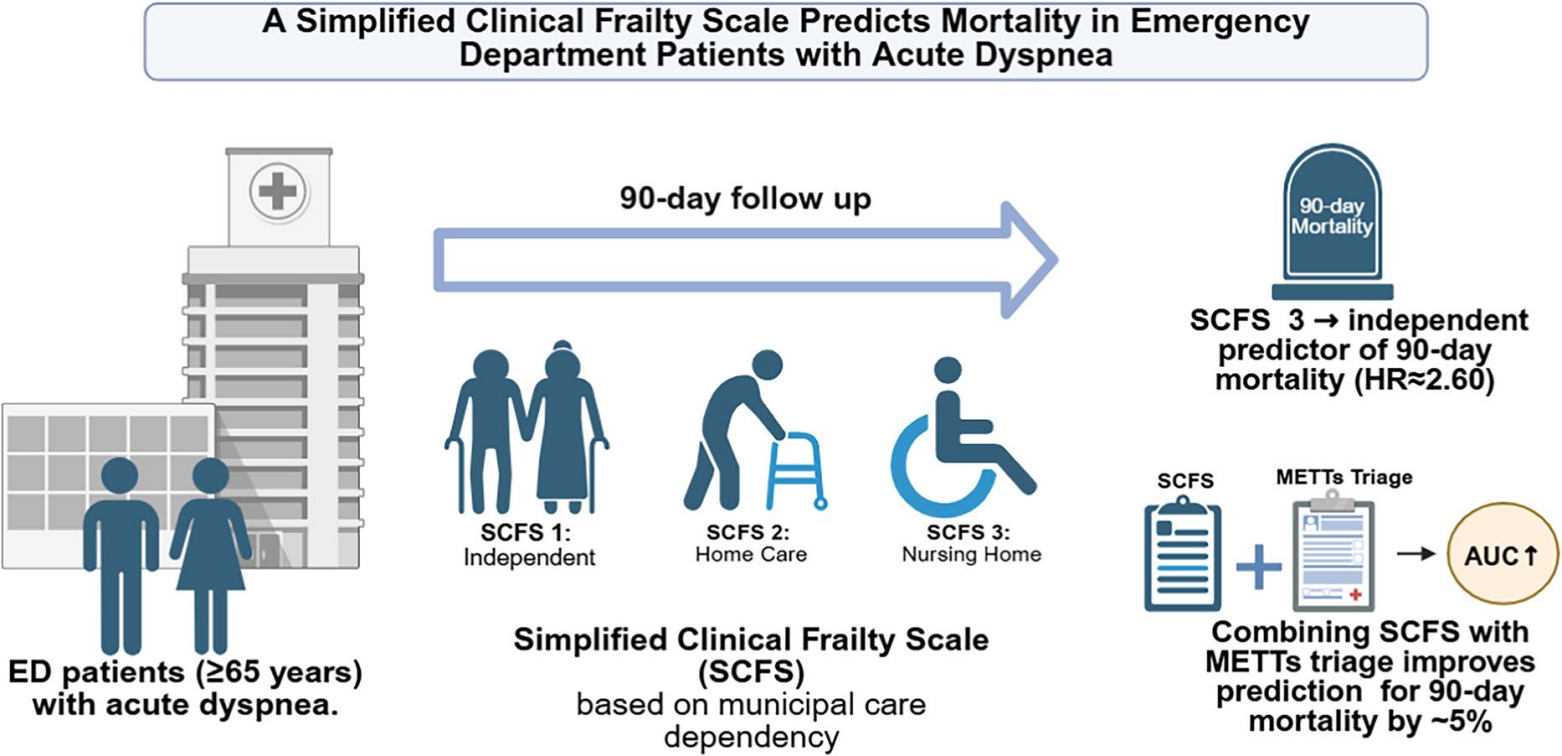

**Supplementary Information:**

The online version contains supplementary material available at 10.1007/s11357-025-01864-7.

## Introduction

Acute dyspnea is one of the most common and challenging presentations in the emergency department (ED) [1], often signifying a high-risk state that requires prompt assessment. Patients presenting with dyspnea span elderly patients, diverse demographics, and comorbidities, with a significant variation in severity, prognosis, and risk, underscoring the need for rapid and reliable identification of those at heightened risk of adverse outcomes [1]. Triage systems, such as the Medical Emergency Triage and Treatment System (METTS) used in Sweden, are commonly employed in the ED to stratify these patients [2]. However, these systems have shown limited accuracy in older adults [2], emphasizing the need for a more effective and user-friendly stratification tool for this patient population.

Aging is characterized by diminished physiological homeostasis and is associated with increased hospitalization and mortality [3]. Closely related to aging, frailty represents an accelerated decline in the body’s regulatory mechanisms, increasing vulnerability to minor stressors and resulting in outcomes such as cognitive decline, disability, greater dependency, and increased revisits and admissions to the ED [3, 4].

One of the most commonly used clinical definitions of frailty is the physical frailty phenotype by Fried and colleagues. This definition is based on criteria related to reduced physical reserves: unintentional weight loss (10 pounds or 4.5 kg in the past year), exhaustion (low energy and self-reported exhaustion), weakness (by grip strength), slowness (by walking speed), and reduced physical activity [5]. A pre-frail stage, in which one or two criteria are present, identifies a subset at high risk of progressing to frailty. The Clinical Frailty Scale (CFS) was developed to provide clinicians with an easily applicable tool to stratify older adults according to their level of vulnerability [6]. The CFS has been validated and was comparable to the Frailty Phenotype in identifying frailty status without complex measurements such as grip strength or gait speed [7]. CFS is particularly useful in the ED and Intensive Care Unit (ICU) for predicting patient outcomes, including hospitalization, mortality, and those in need of urgent care [8–12].

Despite its utility, the CFS has limitations, including its subjective nature, complexity, and inability to identify frail patients with specific diseases [13–15]. The feasibility of applying the CFS in the ED is also questioned [15].

This study was conducted using the ADYS cohort, comprising unselected patients presenting to the emergency department with acute dyspnea. This clinical population is typically older, frequently multimorbid, and often exhibits frailty and clinical vulnerability.

Frailty was based on the patient's level of independence and need for assistance with activities of daily living (ADLs) (using available data on municipal care services) as classified in the original CFS and referred to as the SCFS. SCFS was categorized into three levels:

- **SCFS Group 1 (non-frail):** No municipal care services
- **SCFS Group 2 (moderately frail):** Receiving home care services
- **SCFS Group 3 (severely frail):** Living in nursing homes or short-term care facilities

These SCFS levels were considered to correspond to the following ranges of the original CFS:

- **CFS levels 1–4:** Independent, categorized as non-frail
- **CFS levels 5–6:** Requiring some assistance, categorized as moderately frail
- **CFS levels 7–9:** Dependent, categorized as frail

The rationale for using a three-category model rather than the full nine-level scale is based on the realities of acute care. In the ED, clinicians often have limited time and access to patient history when assessing critically ill individuals, making detailed frailty classification challenging. However, it is usually feasible to form a reasonably accurate impression of whether a patient is non-frail (CFS ≤ 4), moderately frail, or severely frail (CFS ≥ 7), even in a time-pressured environment. This simplified approach aims to strike a balance between clinical relevance and real-world feasibility.

As shown previously for the CFS, we hypothesized that SCFS would be associated with 90-day mortality and hospitalization.

## Methods

### Study design and population

**Fig. 1:**
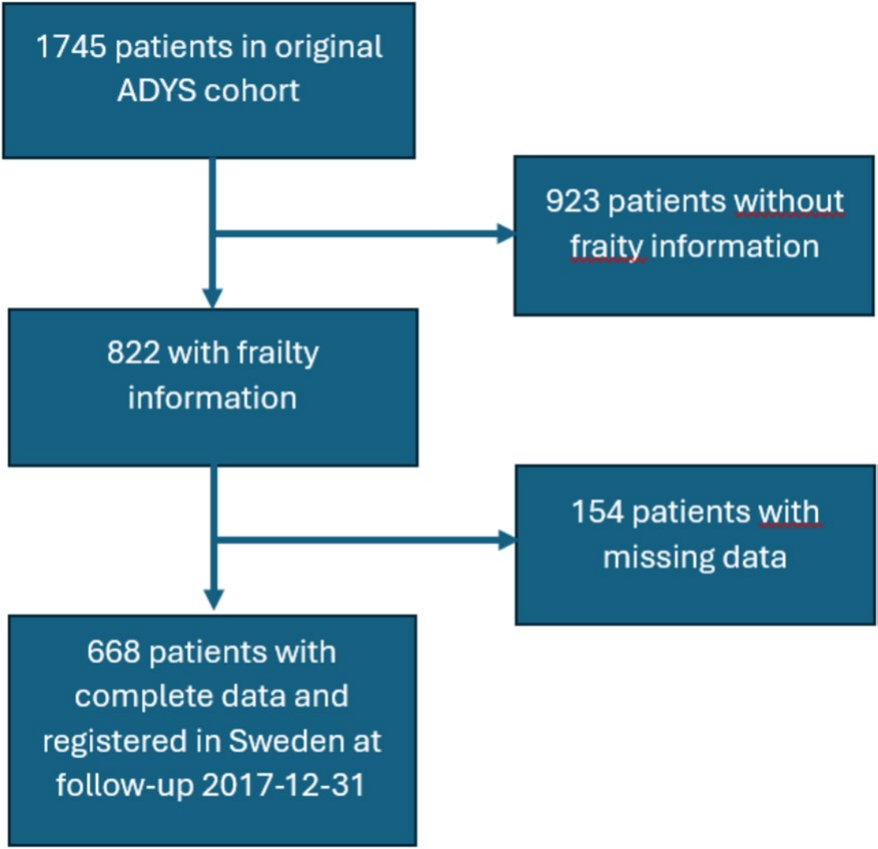
Flow chart of the cohort

Data were obtained from the Acute Dyspnea Study (ADYS) cohort [16]. ADYS included a total of 1,745 patients aged 18 years and older who presented to the emergency department (ED) with dyspnea between March 2013 and January 2019. Patients were included by research nurses on weekdays between 8:00 AM and 5:00 PM at the ED of Skåne University Hospital in Malmö, after obtaining informed consent. Patients in critical condition or with reduced consciousness were not included. After inclusion, patients were interviewed by a research nurse using a standardized and approved questionnaire covering health status, medications, symptoms, and social circumstances. The presence of 22 predefined comorbidities relevant to dyspnea was assessed and confirmed through hospital records (Supplementary Table 1). Patients were triaged according to the Medical Emergency Triage and Treatment System (METTS). The ADYS dataset contained information on municipal care and housing only for the first 822 patients. After excluding 154 individuals due to missing data, the final sample included in this study consisted of 668 patients (Fig. 1).

The METTS triage system originally includes five clinical priority levels in ascending order of urgency: blue (lowest priority – not life-threatening), green, yellow, orange, and red (highest priority – life-threatening). However, due to local triage routines, the blue category was not used in the triage of patients in this study. Therefore, green represented the lowest triage level applied. The severity of dyspnea was graded by research nurses for all patients using the New York Heart Association (NYHA) functional classification of heart failure, ranging from class I to IV [1].

Patients were categorized into three SCFS levels, each reflecting a degree of frailty:

- SCFS 1: Not frail patients with no need for municipal care services
- SCFS 2: Patients with municipal care services, including home care
- SCFS 3: Patients with residence in a short-term care facility or nursing home

The date of death was obtained from the Swedish Cause of Death Register, with mortality data available up to December 31, 2017. Data on healthcare revisits within 12 months after the index date were retrieved from the National Board of Health and Welfare’s inpatient register.

### Statistics

Categorical variables were displayed as frequencies and statistically tested with Pearson’s chi-square test. Continuous variables were displayed as means (and standard deviations, SD), or medians (and interquartile range, IQR). For comparison between the two groups, Mann–Whitney’s U-test or two-tailed t-test was used, depending on the distribution of the variable between groups as assessed by skewness, kurtosis, or inspection of the histogram.

Cox proportional hazards regression was used to estimate hazard ratios (HRs) with 95% confidence intervals (CIs) for 90-day mortality. We conducted both partially adjusted and multivariable analyses to assess the independent association between frailty and mortality.

Logistic regression with odds ratios (ORs) was used to calculate the predicted probability of combined independent variables for the outcomes of hospitalization. The probabilities from the Cox and logistic regressions were then incorporated into receiver operating characteristic (ROC) curves to assess predictive performance. ROC analyses were performed using the R package *pROC*, and DeLong’s test was used to compare the area under the curve (AUC) between two variables.

To control for potential confounding, we included a set of clinically relevant covariates known to influence short-term outcomes in older adults presenting to the emergency department:

- Body Mass Index (BMI): to account for undernutrition or obesity, both of which may influence prognosis and interact with frailty.
- C-reactive Protein (CRP): as a marker of systemic inflammation and acute illness severity.
- Comorbidity burden (> 3 comorbidities): to distinguish frailty from multimorbidity.
- Hospital readmissions (> 3 within 12 months): as an indicator of unstable health and recent healthcare utilization.
- METTS triage level: to adjust for the severity of the acute condition at ED arrival.

We used stepwise multivariable Cox regression to examine the association between SCFS and 90-day mortality. Model A adjusted for age, sex, and BMI; Model B additionally adjusted for indicators > 3 comorbidities, and > 3 hospital revisits; Model C further added METTS triage level; and Model D further added CRP. A forest plot was generated using the R packages *meta*, *survival*, and *forestplot*.

In the Cox regression analysis, statistical significance was set at p < 0.01 due to multiple testing. For all other analyses, the significance threshold was p < 0.05. Kaplan–Meier survival curves were generated using the R packages *survival* and *survminer*. All statistical analyses were performed using IBM SPSS Statistics, version 30.0 (IBM Corp., Armonk, NY, USA).

## Results

### Cohort characteristics

Table 1 summarizes the demographic and clinical characteristics of the patients, stratified by frailty status. Of the 668 patients with documented SCFS groups, SCFS 1 comprised 64.8% of the cohort, while SCFS groups 2 and 3 accounted for 35.2%. A statistically significant association was found between SCFS and gender, with a higher prevalence of SCFS group 2 and 3 among female patients (P = 0.007). Frail patients exhibited a significantly higher rate of hospitalization (P < 0.001), elevated 90-day mortality (P < 0.001), and increased levels of CRP (P = 0.034). They also had more prescribed medications (P < 0.001), were more likely to have more than three comorbidities (P < 0.001), and were more frequently triaged as orange or red by METTS, indicating greater clinical urgency (P < 0.001). In this cohort, the most prevalent comorbidities were chronic heart failure (CHF; 36.4%), coronary artery disease (CAD; 34.6%), atrial fibrillation (AF; 32.0%), and chronic obstructive pulmonary disease (COPD; 31.6%). Compared with non-frail patients (SCFS 1), frail patients (SCFS 2–3) had a significantly higher burden of cardiovascular and cerebrovascular conditions, including CAD, CHF, AF, stroke, and hypertension, along with COPD, infections, renal disease, cancer, anemia, and dementia (all *P* < 0.05; see Table 1). Asthma, pulmonary embolism, obesity, and diabetes did not differ by frailty status.

**Table 1:** Dyspnea-related patient characteristics in the emergency department: a comparison between non-frail and frail patients; n = 668

Variable	All	SCFS group 1 (Non-Frail)	SCFS group 2 + 3 (Frail)	p-value
	**668**	**433(64.8%)**	**235 (35.2%)**	
Age, years, Mean (± SD)	72.9 ± 16.1	67.7 (± 16.2)	82.6 (± 10.3)	< 0.001a
Female, Gender, n (%)	369 (55.2)	222 (51.3)	146 (62.1)	0.007 b
**BMI (kg/m**^**2**^**)**				0.327 b
< 18.5, n (%)	30 (4.6)	17 (4)	13 (5.7)	
18.6–24.9, n (%)	257 (39.2)	163 (38.2)	94 (41.2)	
25.0- < 30, n (%)	206 (31.5)	135 (31.6)	71 (31.1)	
> = 30.0, n (%)	162 (24.7)	112 (26.2)	50 (21.9)	
Total number of medications, Median (IQR)	6 (3–9)	5 (2–8)	8 (6–12)	< 0.001 c
CRP, Median (IQR)	10 (3.6–31)	8.4 (3.35–29)	12 (5–35)	0.034 c
**Outcomes**				
Death within 30 days, n (%)	45 (6.7)	20 (4.6)	25 (10.6)	0.003 b
Death within 90 days, n (%)	88 (13.2)	39 (9)	49 (20.9)	< 0.001 b
Hospitalization, n (%)	408 (61.2)	230 (53.1)	178 (76.1)	< 0.001 b
More than 3 Re-visits	106 (15.9)	65 (15)	41 (17.4)	0.411 b
More than 3 comorbidities	335 (50.1)	169 (39)	166 (70.6)	< 0.001 b
**Triage/METTS**				< 0.001 b
Green, n (%)	40 (6)	35 (8.1)	5 (2.1)	
*Yellow*, n (%)	310 (46.5)	230 (53.2)	80 (34)	
*Orange*, n (%)	234 (35.1)	129 (29.9)	105 (44.7)	
*Red*, n (%)	83 (12.4)	38 (8.8)	45 (19.1)	
**Comorbidities**				
Coronary artery disease, n (%)	231 (34.6)	136 (31.4)	95 (40.4)	0.019 b
Chronic heart failure, n (%)	243 (36.4)	125 (28.9)	118 (50.2)	< 0.001 b
Atrial fibrillation disease, n (%)	214 (32)	106 (24.5)	108 (46)	< 0.001 b
Hypertension, n (%)	321 (48.1)	188 (43.4)	133 (56.6)	0.001 b
Stroke, n (%)	76 (11.4)	35 (8.1)	41 (17.4)	< 0.001b
Asthma, n (%)	75 (11.2)	50 (11.5)	25 (10.6)	0.722 b
Pulmonary embolism, n (%)	86 (12.9)	49 (11.3)	37 (15.7)	0.103 b
Chronic obstructive pulmonary disease, n (%)	211 (31.6)	122 (28.2)	89 (37.9)	0.010 b
Restrictive lung disease, n (%)	34 (5.1)	20 (4.6)	14 (6)	0.452 b
Infections, n (%)	201 (30.1)	102 (23.6)	99 (42.1)	< 0.001 b
Anemia, n (%)	134 (20.1)	62 (14.3)	72 (30.6)	< 0.001 b
Cancer, n (%)	125 (18.7)	69 (15.9)	56 (23.8)	0.012 b
Obesity, n (%)	148 (22.2)	102 (23.6)	46 (19.6)	0.237 b
Diabetes, n (%)	128 (19.2)	76 (17.6)	52 (22.1)	0.151 b
Renal disease, n (%)	70 (10.5)	32 (7.4)	38 (16.2	< 0.001 b
Dementia, n (%)	21 (3.1)	7 (1.6)	14 (6)	0.002 b

P-value was tested by a) Two-tailed independent t-test b) Chi-square test c) Mann–Whitney U test. Abbreviation: Standard Deviation (SD), Body Mass Index (BMI), Interquartile range (IQR), Medical Emergency Triage and Treatment System (METTS)

### Outcomes: 90-day mortality and hospitalization

**Fig. 2:**
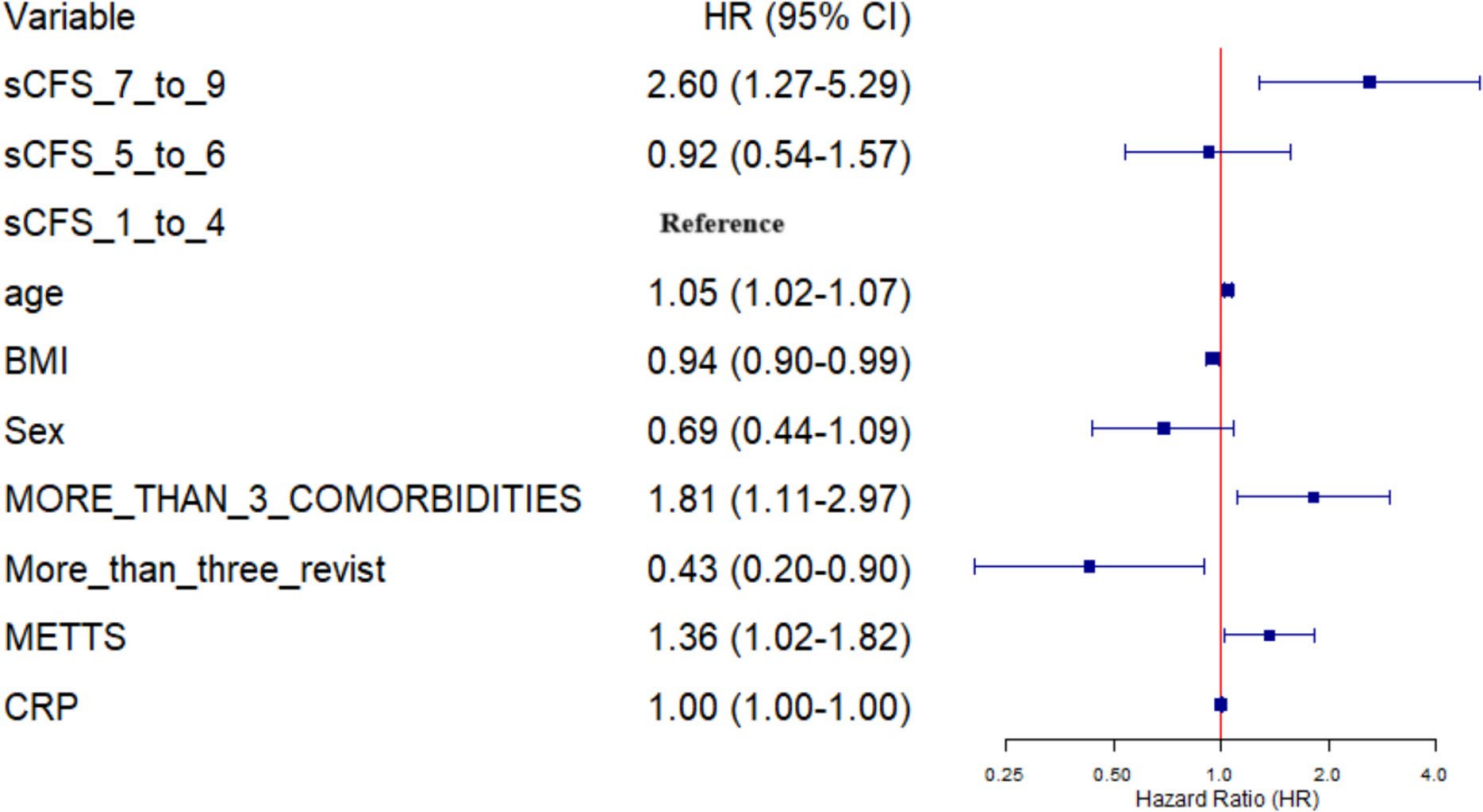
Forest plot of HR for variables associated with 90-day mortality corresponding to Model D of Table 3. This forest plot presents the HR (blue boxes), corresponding to 95% CI (horizontal blue line), for the association of selected variables with 90-day mortality, where the red vertical line corresponds to an HR of 1

In univariate Cox regression analysis, SCFS groups 2 and 3 were significantly associated with the hazard of 90-day mortality, with SCFS group 1 as a reference (Table 2). After adjusting for confounders, patients with an SCFS group 3 had a 2.60-fold higher hazard of 90-day mortality compared to those with an SCFS group 1 (HR = 2.60, 95% CI: 1.27–5.29, P = 0.009), independent of age, BMI, gender, comorbidities, hospital revisit, METTS score, and CRP levels (Table 3). The forest plot displayed the adjusted variables that were associated with the hazard of 90-day mortality (Fig. 2). Kaplan–Meier survival analysis further demonstrated that patients in the SCFS group 3 had the highest hazard of 90-day mortality (P < 0.001) (Supplementary Figure S1).

**Table 2:** Unadjusted relative risks of 90-day mortality derived from Cox regression models; *n* = 668

Predictor	HR	95% CI	P-value
SCFS group 1			
SCFS group 2	1.99	1.25–3.17	0.004
SCFS group 3	4.97	2.78–8.90	< 0.001

Results are displayed as hazard ratios (HR) corresponding to 95% confidence intervals (CI), and P-value tested whether the 95% for HR contains 1

**Table 3:** The association between SCFS group 3 and the relative risk of mortality within 90 days (Multivariate Cox regression models); *n* = 668

Predictor	HR	95% CI	P-value
Model A, SCFS group 1 reference			
SCFS group 2	1.04	0.62–1.74	0.878
SCFS group 3	2.79	1.41–5.54	0.003
Model B, SCFS group 1 reference			
SCFS group 2	0.97	0.57–1.61	0.908
SCFS group 3	2.76	1.38–5.50	0.004
Model C, SCFS group 1 reference			
SCFS group 2	0.91	0.53–1.54	0.716
SCFS group 3	2.44	1.20–4.97	0.014
Model D, SCFS group 1 reference			
SCFS group 2	0.92	0.54–1.57	0.766
SCFS group 3	2.60	1.27–5.29	0.009

Model A (SCFS + age, BMI, gender), Model B: Model A +  > 3 comorbidities +  > 3 revisits, Model C: Model B + METTS, Model D: Model C + CRP

**Fig. 3:**
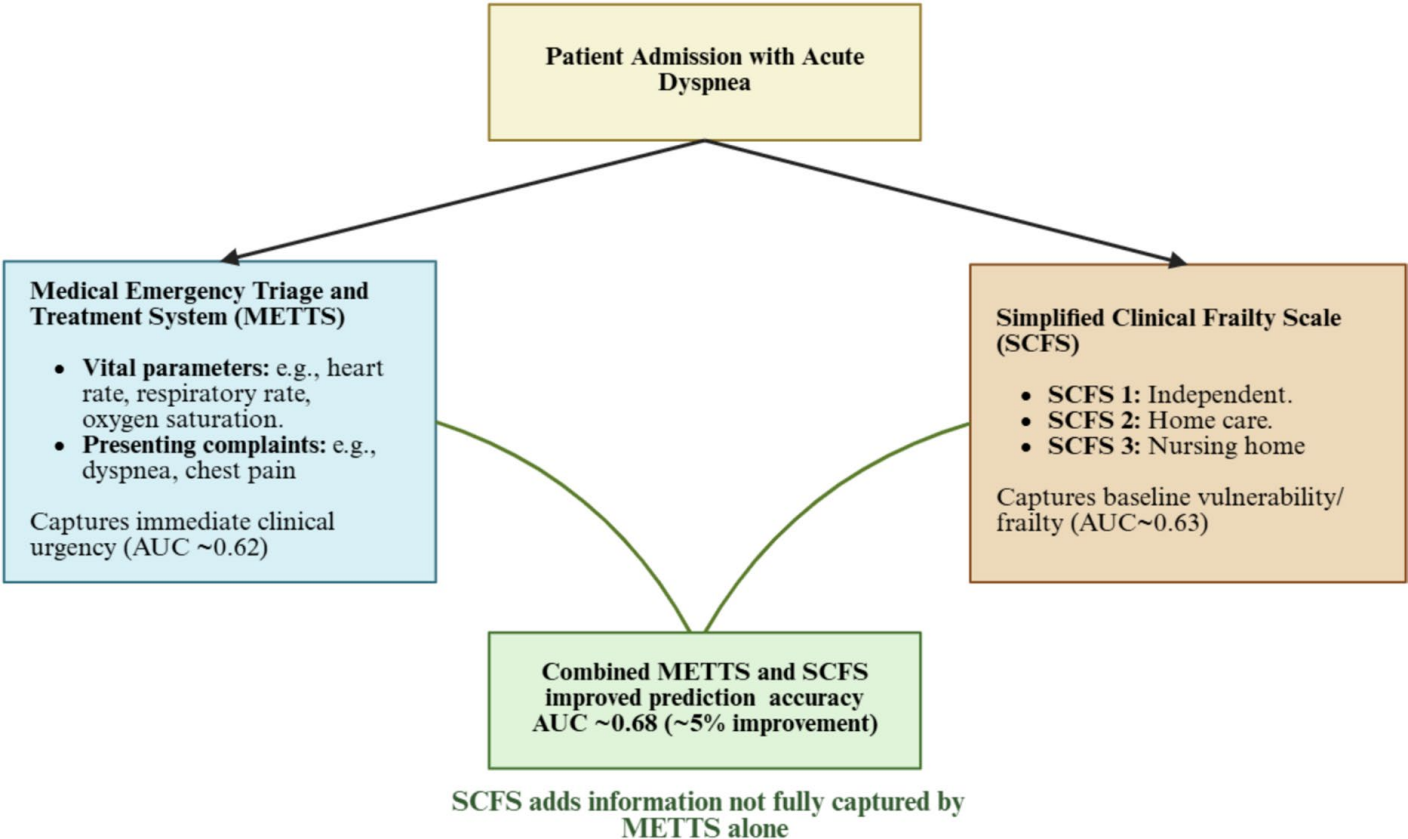
Schematic illustration of the complementary predictive value of SCFS, METTS, and their combination for 90-day mortality. The SCFS and METTS individually demonstrate moderate discriminatory ability (AUC ~ 0.62–0.63) for 90-day mortality among patients presenting with acute dyspnea. When combined, these tools provide incremental predictive utility, yielding an approximate 5% increase in overall prediction accuracy (AUC ~ 0.68) compared to either measure alone

Regarding mortality risk in the ROC analysis, adjusting for SCFS (P < 0.001) as a covariate yielded an area under the curve (AUC) of 0.631, indicating moderate discriminative ability (Supplementary Table S1). DeLong’s test showed that SCFS and METTS have comparable predictive accuracy for 90-day mortality (P = 0.791, Supplementary Table S2). However, when the predictive performance of a combined SCFS-METTS model for 90-day mortality was evaluated against the individual models, the combined model achieved an AUC of 0.681, significantly outperforming the SCFS model alone (P = 0.015, 95% CI for the AUC difference: −0.078 to −0.008) (Supplementary Figure S2 Model A, Fig. 3). Furthermore, the combined SCFS-METTS model showed significant improvement over the METTS model alone (P = 0.032, 95% CI for the AUC difference: −0.102 to −0.005) (Supplementary Figure S2 Model B, Fig. 3). These findings suggest that integrating SCFS with METTS enhances the predictive accuracy for 90-day mortality in this population (Fig. 3).

Univariate logistic regression indicated a significant association between SCFS categories and the odds of hospitalization (Supplementary Table S3). After controlling for potential confounders, patients in SCFS group 3 had a 2.57-fold increase in the odds of hospitalization compared to those with SCFS group 1 (OR = 2.57, 95% CI: 1.11–6.71, P = 0.037), after adjusting for age, gender, BMI, comorbidities, and readmission status (Supplementary Figure S3, Model A, and Table 4). However, this association lost statistical significance after further adjustment for METTS (Supplementary Figure S3, Model B, and Table 4). ROC curve analysis showed that SCFS alone had an AUC of 0.610 (P < 0.001) (Supplementary Table S4).

**Table 4:** Adjusted models of multivariable logistic regression in which SCFS predicting hospitalization

Predictor	OR	95% CI	P-value
**Model A**, SCFS group 1 Reference			
SCFS group 2	1.58	1.04–2.41	0.034
SCFS group 3	2.87	1.19–6.96	0.019
**Model B**, SCFS group 1 Reference			
SCFS group 2	1.43	0.93–2.21	0.104
SCFS group 3	2.57	1.11–6.71	0.037
**Model C**, SCFS group 1 Reference			
SCFS group 2	1.12	0.71–1.79	0.620
SCFS group 3	1.67	0.68–4.58	0.287

Model A (age, gender, BMI), Model B (Model A and > 3 comorbidities +  > 3 revisit), Model C (Model B and METTS)

## Discussion

### Main findings

In this cohort of emergency department (ED) patients admitted for dyspnea, we found that the SCFS was independently associated with 90-day mortality, even after adjusting for key clinical variables. Moreover, combining SCFS with the triage tool METTS significantly improved predictive accuracy.

### Conceptual complexity and frailty definitions

Frailty is a complex, multidimensional clinical syndrome characterized by reduced physiological reserve and increased vulnerability to stressors. Despite growing recognition of frailty as a key predictor of adverse outcomes, there is no universally accepted definition or assessment tool. International guidelines from the International Conference on Frailty and Sarcopenia Research (ICFSR) [17] and the British Geriatrics Society [18] emphasize its multidimensional nature, spanning physical, cognitive, psychological, and social domains [19]. Conceptual models such as the Fried phenotype [20] and the Frailty Index (FI) by Rockwood et al. [21], reflect this complexity and contribute to variability in frailty classification across studies and clinical settings. The Clinical Frailty Scale (CFS) [22], the simplified clinical tool derived from the FI, is widely used in Swedish healthcare [23], but has limitations. It relies on subjective clinical judgment and often requires knowledge of a patient's functional status, which may not be available in emergency settings [15].

The Clinical Frailty Scale has been validated and was comparable to the Frailty Phenotype for identifying frailty status without requiring complex measurements, such as grip strength or gait speed [24]. The Clinical Frailty Scale has been used in the clinical setting, and increased frailty according to CFS was a strong predictor of in-hospital mortality, increased length of hospitalization, new nursing home placement, early readmission, and functional impairment [25, 26]. Frailty as measured by CFS was associated with the outcome after heart surgery [27, 28], geriatric trauma [29] after elective and acute surgery [30] in patients with pneumonia [31], heart failure [32], renal failure [33], and liver cirrhosis [34], and in patients admitted to intensive care [35]. Additionally, CFS has been successfully tested in the emergency department setting [36–39] and proven to be of clinical value.

### Our interpretation of the findings

In 2020, the Clinical Frailty Scale (CFS) was revised and updated to version 2.0 [40] to enhance its applicability as a triage tool and to provide clearer guidance for clinical decision-making regarding care goals and further treatment. The updated scale maintains a structured gradation of frailty from level 1 (very fit) to level 9 (terminally ill), with levels 1 to 3 representing individuals without impairments in either instrumental or personal activities of daily living (ADLs).

In contrast, from level 4 onwards, there is a progressive increase in limitations related to mobility, functional capacity, and cognitive abilities. Level 4 describes individuals who are not fully dependent but show clear limitations in daily function, particularly in more demanding tasks such as housework activities, often among the first to be affected. At level 6, now termed"Living with Moderate Frailty"(formerly"Moderately Frail"), dependency extends into intermediate ADLs, including activities such as bathing. By level 7,"Living with Severe Frailty"(previously"Severely Frail"), individuals experience substantial dependency even in basic personal ADLs, indicating a marked progression of frailty and loss of autonomy.

SCFS levels 2 and 3 represent a notable shift in dependency compared to SCFS level 1, with the key distinction being the degree of dependency in personal activities of daily living (ADLs). In the original Clinical Frailty Scale (CFS), it is estimated that approximately 80% of patients can be accurately categorized into a single frailty level. In cases where two levels appear equally applicable, the recommendation is to assign the higher frailty level [40].

The interpretation of ADL dependency can vary significantly between individuals, as specific tasks may be more or less demanding depending on personal and contextual factors. Consequently, we expect a degree of heterogeneity in frailty status within our study population, and some individuals might have been more appropriately classified under a different level.

Unlike the original CFS, the SCFS is structured exclusively around the actual degree of home care required. This design aims to reduce inter-rater variability by anchoring frailty classification to observable, care-related criteria, potentially enhancing consistency and minimizing subjective interpretation when estimating frailty levels.

Our results align with previous research demonstrating associations between frailty and outcomes such as mortality, rehospitalization, and increased healthcare utilization [41–44]. While the discriminatory ability was modest (AUC 0.63–0.68), this is consistent with prior studies in older, multimorbid populations, where short-term prognostication remains challenging. Nonetheless, these findings support the clinical utility of frailty measures, which may yield meaningful prognostic insights even with moderate predictive performance [22, 45]. Furthermore, integrating frailty assessments into triage systems has been shown to enhance the prediction of ICU admissions and mortality [46]. Mowbray et al. have highlighted the complementary value of combining frailty assessment with triage tools in emergency care settings [47]. In our cohort, frail patients (35.2%) were more likely to be female and to present with multiple comorbidities, elevated CRP levels, and polypharmacy patterns that mirror those described in prior research on frailty, systemic inflammation, and medication burden [48–50].

### Clinical implications

Our findings indicate that the SCFS enables early identification of vulnerable patients who may benefit from individualized care planning. In time-critical emergency settings, the simplified three-level SCFS offers a more practical alternative to the full 9-point CFS. SCFS allows for automated frailty estimation using routinely collected health system data within existing digital infrastructure. Although METTS triage predominates in predicting immediate hospitalization risk, SCFS adds complementary prognostic information, highlighting patients at higher risk of downstream resource needs (e.g., geriatric consults, post-acute services) that METTS alone does not capture. Integrating SCFS alongside METTS can therefore guide both acute management and longer-term care planning.

To support real-world implementation, the SCFS could be integrated into electronic health record (EHR) systems as automated prompts during triage or initial assessment, enabling routine frailty screening without introducing significant delays. Its use by triage nurses or clinicians as part of the standard triage process may also be feasible [51].

### Strengths and limitations

This study has several notable strengths. This is a cohort study with follow-up for mortality, allowing for temporal analysis of outcomes based on baseline characteristics. The large sample size increases statistical power and enhances the reliability of the findings. The use of real-world data from routine clinical care strengthens the study's external validity and practical relevance. The SCFS measure, based on existing patient records (e.g., home care status, nursing home residence), was easily obtainable and required no bedside assessment, making it well-suited for integration into emergency department (ED) workflows. Finally, by evaluating frailty in the context of high-demand, time-sensitive ED settings, the study highlights the potential for rapid, low-burden risk stratification in acute care.

#### This study has several limitations

First, it was conducted at a single center, which may limit the generalizability of the findings, as the patient population and clinical routines may differ from those in other settings. Additionally, the METTS triage tool, as implemented in Sweden, serves as a risk stratification tool similar to other triage tools, including, for example, the Emergency Severity Index (ESI) or the Manchester Triage System (MTS). However, there are differences in structure and clinical application across different triage algorithms which may affect outcomes. Consequently, caution is advised when attempting to generalize or replicate these findings in healthcare settings that utilize alternative triage tools. Second, the use of the categorical SCFS variable restricts a more nuanced analysis of frailty severity and its associations with outcomes. Another limitation of this study is that the SCFS does not distinguish pre-frail patients from non-frail and frail individuals. Pre-frail patients (as defined by the original CFS as score 4) are considered vulnerable and at increased risk of further decline in health and function. Compared to frail patients, pre-frail patients are often younger and less well studied, despite their high prevalence. This may be due to the difficulty in accurately identifying this intermediate state, particularly in the ED. Nonetheless, we believe that recognizing prefrail patients in the ED is important to implement achievements to prevent further deterioration [52]. Considering the moderate discriminatory accuracy of SCFS; SCFS it should not be relied upon as a stand-alone prognostic tool, but rather as one component of a broader, multimodal clinical assessment. Third, although we adjusted for multiple potential confounders, unmeasured factors—such as socioeconomic status, cognitive function, or access to follow-up care—may have influenced the observed associations. There is also a potential selection bias: only patients who were sufficiently well to communicate and provide information at the time of emergency department presentation could be included. As a result, more severely ill individuals—such as those with reduced consciousness, acute confusion, or cognitive impairment—may have been underrepresented. This limitation could affect the applicability of the findings to the most vulnerable subgroup of older patients. Finally, although the SCFS is an adapted version of the original CFS, it still requires validation against the original CFS to confirm its accuracy and reliability.

## Conclusion

SCFS was found to be independently associated with 90-day mortality. When combined with METTS, the SCFS significantly improved the discriminative ability for predicting 90-day mortality. These findings underscore the utility of SCFS as a practical alternative to the traditional 9-point CFS, especially in high-demand clinical settings such as emergency departments, where rapid risk stratification is critical.

## Supplementary Information

Below is the link to the electronic supplementary material.

Supplementary file1 (DOCX 137 KB)

## Data Availability

The datasets generated during and/or analyzed during the current study are available from the last author on reasonable request.

## Abbreviations

ADYS: Acute Dyspnea Study
AUC: Area Under Curve
BMI: Body Mass Index
CHF: Congestive Heart Failure
CRP: C-Reactive Protein
ED: Emergency Department
ICD-10: International Classification of Diseases version 10
METTS: Medical Emergency Treatment and Triage System
ROC: Receiver Operating Characteristic
SD: Standard Deviation
SCFS: Simplified Clinical Frailty Scale
