# Supplementary material for: A simplified clinical frailty scale predicts mortality in emergency department patients with acute dyspnea
Source: GeroScience. 2025 Sep 11;48(3):4011–23. doi: 10.1007/s11357-025-01864-7 (PMC13356002; doi:10.1007/s11357-025-01864-7)
Supplement: Supplementary file 1 — Supplementary file1 (DOCX 137 KB) [file 11357_2025_1864_MOESM1_ESM.docx]

**Supplementary files**

**Supplementary Table S1. ROC: AUC analysis regarding 90-day mortality.**  The variables are adjusted as covariates in the ROC model.

| 90-day mortality (n=82) | | |
| --- | --- | --- |
| Variables | AUC | P-value |
| METTS | 0.619 | <0.001 |
| BMI | 0.592 | 0.007 |
| Age | 0.710 | <0.001 |
| Hospitalization | 0.65 | <0.001 |
| Total number of Medications | 0.63 | <0.001 |
| CRP | 0.66 | <0.001 |
| sCFS | 0.63 | <0.001 |
| sCFS + METTS | 0.67 | <0.001 |
| sCFS + Age | 0.72 | <0.001 |
| sCSF + Age + METTS | 0.73 | <0.001 |

Results are displayed by Area Under the Curve (AUC), with P-value testing whether the AUC is greater than 0.5 (indicating no discrimination). The positive outcome for 90-day mortality is 82 instead of 88, as sCFS is adjusted with a covariate that misses information for some patients. This results in a different AUC compared to evaluating the whole positive outcome (n=88).

# **Supplementary Table S2. Delong’s test for comparing the AUC between the sCFS-METTs model with SCFS only and METTs only for 90-day mortality.**

| **Varible** | **AUC** | **95% CI for AUC differences** | **P-value** |
| --- | --- | --- | --- |
| **METTs-sCFS** | **0.68** | -0.078 to -0.008 | 0.015 |
| **sCFS** | **0.63** |  |  |
|  | | | |
| **METTs-sCFS** | **0.68** | -0.102 to -0.005 | 0.032 |
| **METTs** | **0.63** |  |  |
|  | | | |
| **sCFS** | **0.64** | **-0.066 to 0.086** | **0.791** |
| **METTs** | **0.62** |  |  |

Results display the differences between the AUC of two variables, with the 95% CI of the AUC differences, tested by P-value. The positive outcome is 87 instead of 88, as METTS lacks information about one patient.

**Supplementary Table S3. Univariate logistic regression predicting hospitalization.**

| Predictor | OR | 95% CI | P-value |
| --- | --- | --- | --- |
| Frailty score (1-4) |  |  |  |
| Frailty score (5-6) | 2.648 | 1.816-3.861 | <0.001 |
| Frailty score (7-9) | 3.751 | 1.697-8.290 | 0.001 |

Results are displayed as odds ratio (OR) corresponding to a 95% confidence interval (CI), and the P-value tests whether the 95% for OR contains 1.

**Supplementary Table S4. ROC: AUC analysis predicting Hospitalization**

| Hospitalization, Positive outcomes (n=393) | | |
| --- | --- | --- |
| Variables | AUC | P-value |
| METTS | 0.72 | <0.001 |
| BMI | 0.47 | 0.268 |
| Age | 0.68 | <0.001 |
| sCFS | 0.61 | <0.001 |
| Total number of Medications | 0.69 | <0.001 |
| CRP | 0.68 | <0.001 |
| Age + sCFS | 0.69 | <0.001 |
| sCFS + METTS | 0.74 | <0.001 |
| Age + sCFS + METTS | 0.77 | <0.001 |
| METTS + Age | 0.77 | <0.001 |

**Results are displayed by Area Under the Curve (AUC), with P-value testing whether the AUC is greater than 0.5 (indicating no discrimination).**

**Supplementary Figure S1. The Kaplan-Meier curve illustrates the hazard of 90-day mortality of different categories of sCFS.** Log-rank tested the P-value of different categories of sCFS, illustrating a significant difference in the hazard of survival of different categories of sCFS (P-value < 0.001, tested by log-rank test). The graph was generated through the package survival, survminer in R.

**
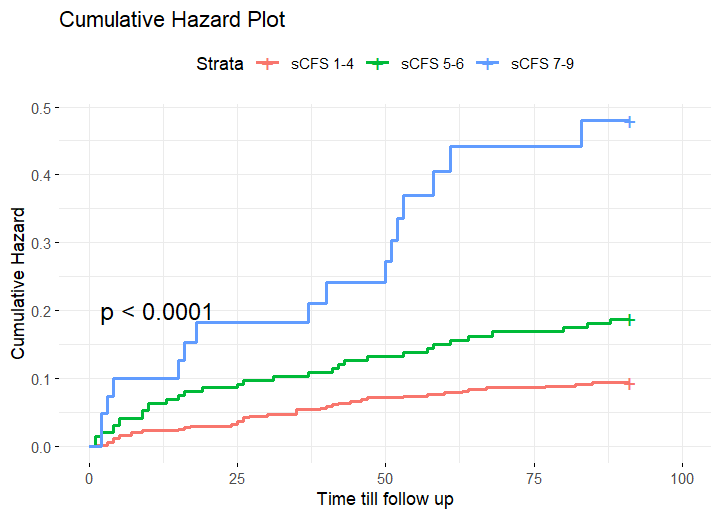
**

**Supplementary Figure S2. ROC Curve Analysis for 90-Day Mortality.** Model A illustrates the comparison of the Area Under the Curve (AUC) with 95% Confidence Intervals (CI) between the sCFS and sCFS-METTS models, with a statistically significant difference (P = 0.015, assessed using DeLong’s test). Model B demonstrates the comparison of the AUC with 95% CI between the METTS and sCFS-METTS models, also showing a statistically significant difference (P = 0.0322, assessed using DeLong’s test). This figure highlights the predictive performance of the combined sCFS-METTS model relative to the individual models.

| **Model A** | **Model B** |
| --- | --- |
| 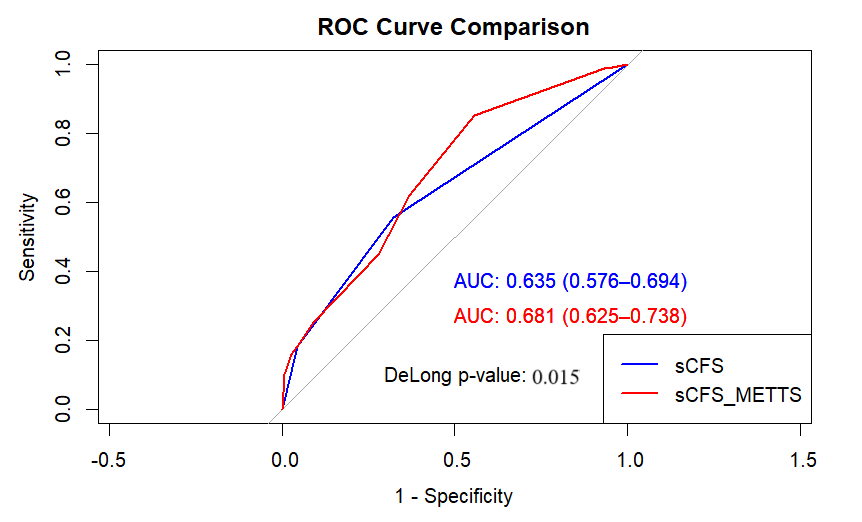 | 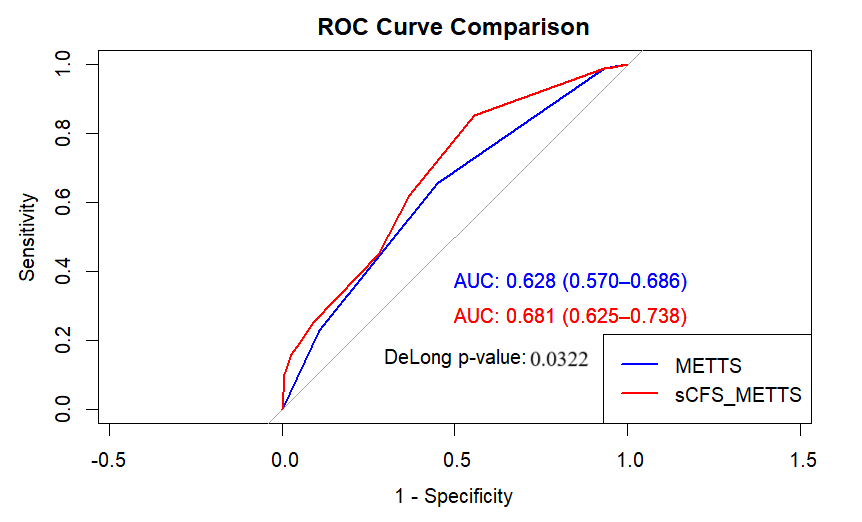 |

**Supplementary Figure S3. Forest plot of OR for variables associated with Hospitalization corresponding to Model B and C of Table 7**. This forest plot presents the OR (blue boxes), corresponding to 95% CI (horizontal blue line), for the association of selected variables with 90-day mortality, where the red vertical line corresponds to an OR of 1. Model A shows the variables adjusted without METTS. Model B shows the variables adjusted with METTS.

| **Model A** | **Model B** |
| --- | --- |
| 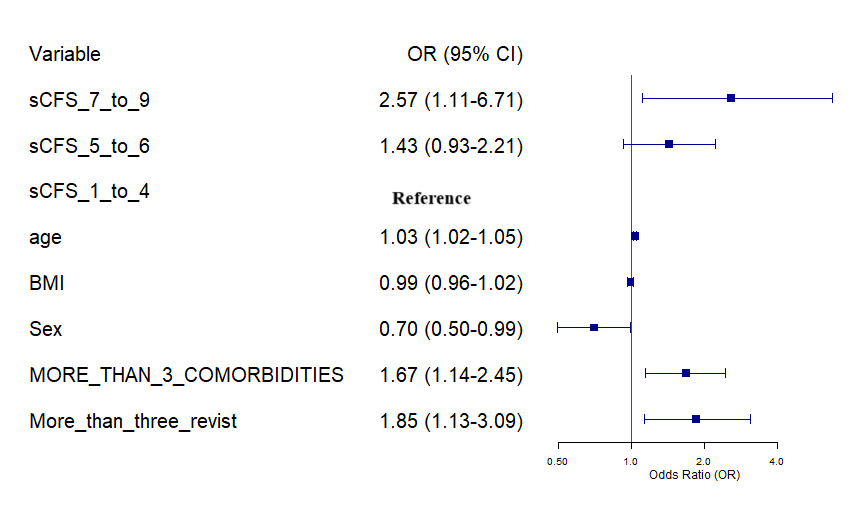 | 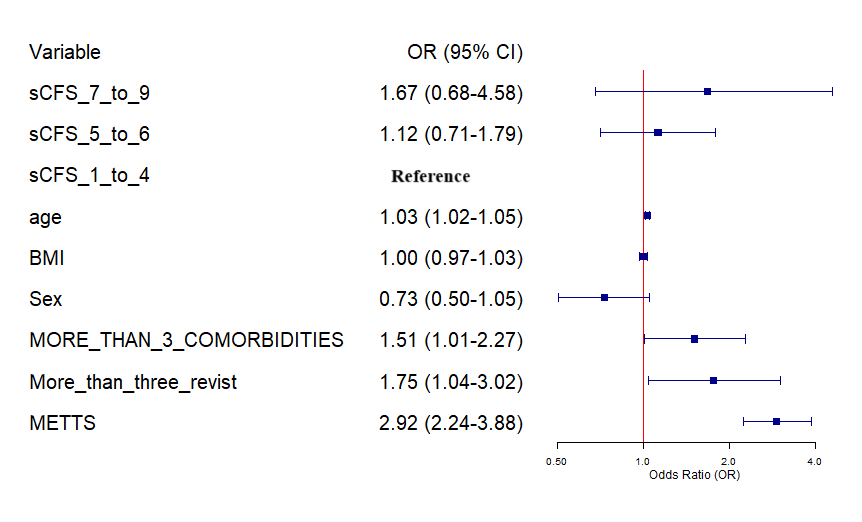 |
